# Adaptive Node Clustering Technique for Smart Ocean under Water Sensor Network (SOSNET)

**DOI:** 10.3390/s19051145

**Published:** 2019-03-06

**Authors:** Mehr Yahya Durrani, Rehan Tariq, Farhan Aadil, Muazzam Maqsood, Yunyoung Nam, Khan Muhammad

**Affiliations:** 1 Department of Computer Science, COMSATS University Islamabad, Attock Campus, Attock 43600, Pakistan; mehryahya@cuiatk.edu.pk (M.Y.D.); rehan@cuiatk.edu.pk (R.T.); farhan.aadil@cuiatk.edu.pk (F.A.); muazzam.maqsood@cuiatk.edu.pk (M.M.); 2 Department of Computer Science and Engineering, Soonchunhyang University, Asan 31538, Korea; 3 Department of Software, Sejong University, Seoul 143-747, Korea

**Keywords:** smart ocean, underwater communication and networks, routing, clustering, optimization, moth flame optimizer, transmission range optimization

## Abstract

Smart ocean is a term broadly used for monitoring the ocean surface, sea habitat monitoring, and mineral exploration to name a few. Development of an efficient routing protocol for smart oceans is a non-trivial task because of various challenges, such as presence of tidal waves, multiple sources of noise, high propagation delay, and low bandwidth. In this paper, we have proposed a routing protocol named adaptive node clustering technique for smart ocean underwater sensor network (SOSNET). SOSNET employs a moth flame optimizer (MFO) based technique for selecting a near optimal number of clusters required for routing. MFO is a bio inspired optimization technique, which takes into account the movement of moths towards light. The SOSNET algorithm is compared with other bio inspired algorithms such as comprehensive learning particle swarm optimization (CLPSO), ant colony optimization (ACO), and gray wolf optimization (GWO). All these algorithms are used for routing optimization. The performance metrics used for this comparison are transmission range of nodes, node density, and grid size. These parameters are varied during the simulation, and the results indicate that SOSNET performed better than other algorithms.

## 1. Introduction

Oceans cover about 71% of the earth’s surface. They have large amount of minerals, oil, vast population of animals and other creatures. Ocean studies has attracted attention for a long time but has received renewed focus in the last decade of the 20th century, when the issues of global warming and climate change surfaced. Smart Ocean is a broad term describing the use of sensors combined with geographic information systems for monitoring the ocean surface [1]. One of the initial efforts in this area is Monterey Bay Aquarium Research Institute (MBARI) Ocean Observing System (MOOS) [2]. This observation system consists of sensor nodes distributed on a wide area throughout the ocean bed. MOOS observatories provide real time data to scientists, which enables them to effectively monitor ocean related phenomena. In addition to the MOOS, Martha’s Vineyard Coastal Laboratory and the New Millennium Observatory [3,4] are other such observatories providing similar kinds of services to scientists. These observatories provide huge amounts of data, but unfortunately the data are not interoperable. To overcome this problem, a smart ocean sensors consortium [1] has developed the PUCK protocol, which makes it easy to exchange information among ocean sensors. Since all the data is acquired from sensor nodes deployed under the sea, studies in smart ocean architecture tend to relate to research on underwater sensor networks. A brief introduction regarding the same is given in the following.

Underwater sensor networks (UWSN) have been a focus of research for many years. They have gained significance in oceanic research and provided data for many applications, such as ocean surface monitoring, disaster prevention, habitat monitoring, and surveillance [5]. In comparison to the terrestrial counterparts, the sensors in UWSN are equipped with an acoustic modem, designed to work in an underwater environment, an antenna for transmission, a pressure gauge, and a bladder apparatus for controlling the depth. These sensors form a sensor equipped aquatic swarm (SEAS), which coordinates with sink nodes at the sea surface. Sensor nodes perform monitoring of the underwater data and send critical data to the sink nodes in a multi-hop fashion. The sink nodes are armed with acoustic and radio communication abilities to receive the data from under water sensors and relay them to the outer world. Like any other sensor network, routing is a critical problem for UWSN [6].

Earlier attempts to develop routing protocols for UWSN were inspired by their terrestrial counterparts. Although similar in nature, the UWSN do have some significant variations from wireless sensor networks (WSN), which make it difficult for the routing protocols developed for WSN to be used as they are in UWSN. One of the main reasons is that the WSN routing protocols are designed for radio frequency (RF) waves, which attenuate significantly under water; hence, RF based sensors are ineffective in UWSN. Sensors in UWSN are equipped with acoustic modems that are tailored to work in underwater environments [7].

Early routing protocols proposed for UWSN were following the classical layered protocol architecture, i.e., the protocols developed for different layers, e.g., transport layer and network layer. However, performance of these protocols was dependent on parameters related to the specific layer. However, these protocols provided poor performance overall. The basic reason for the poor performance of these so called non-cross layer protocols is that they did not consider the energy levels, joint optimization, and other factors that are important in overall performance. Hence the focus of recent research is cross layer routing protocols, which use the information received from various layers [8].

In addition to the above-mentioned problems, there are other significant issues that need to be considered for designing an efficient routing protocol for UWSN. One such issue is high propagation delay. As discussed in [9], the propagation delay of acoustic waves is 200,000 times higher than that of RF waves, which poses a great challenge and high delay. At the same time, due to water currents and extreme weather conditions, the noise in UWSN is very high; noise is introduced because of tides, water movement, and the variations on sea current. Another source of noise is various types of machinery, such as ships, water pumps, and power plants. Thus, removal of noise is a big challenge in designing a routing protocol. Geometric spreading, i.e., the spreading of sound energy, is another problem in UWSN. The reason for geometric spreading is expansion of wave fronts. Another problem introduced by the water currents is change of topology of sensors, which has a great effect on the performance of a routing protocol. On the other hand, the bandwidth of acoustic waves is very low, ranging between one and fifty kHz [10]. Further, the transmission power required in UWSN [11] is significantly higher as compared to terrestrial networks. Another problem is introduced by Doppler spread, which creates significant problems in high speed network as it introduces symbol interference [12]. Finally, sensors are subject to corrosion and fouling in UWSN, which results in failure [13].

The focus of this paper is the design of an efficient routing protocol for UWSN. The crux of the proposed routing protocol, SOSNET, is dividing the UWSN into different clusters and developing an effective dynamic clustering scheme for selection of a cluster head responsible for coordination with sink nodes. The selection of the cluster head will depend on available energy in a sensor node. Once the energy of a cluster head decreases from a given threshold, a new cluster head is selected through an election mechanism. The following are the brief contributions of this paper:

- Optimization of UWSN’s routing by employing the evolutionary algorithms. These algorithms have already been successfully used in other similar network types, such as (Mobile ad hoc Networks) MANETs, vehicular ad hoc networks (VANETs), and (Flying ad hoc Networks) FANETs to name a few. Our proposed technique, SOSNET, is a moth flame optimization-based technique that reduces the overall routing cost and makes the UWSN more efficient.
- SOSNET’s performance has been compared with other state of the art evolutionary algorithms and the results exhibit that the proposed SOSNET provides better results.

The remainder of the paper is organized as follows. Section 2 discusses the related work in this area, Section 3 introduces the key concepts of moth flame optimization, Section 4 provides the detail regarding the proposed algorithm, and Section 5 provides details of the experimental setup. Section 6 provides simulation results and Section 7 concludes the discussion.

## 2. Related Work

As with any sensor network, energy preservation is a major goal for UWSN. Many energy efficient routing protocols have been proposed using the depth information. Depth based routing (DBR) [14] is such a scheme that measures the depth of the sensor and based on this information, data from the sensor, which has a lower depth, is sent to the sink nodes. The information of the remaining energy along with depth information is used in energy efficient depth based routing [15]. To mitigate the issue of flooding proposed in DBR, [16] introduces depth based multi-hop routing, which uses multi-hop transmission for reduction of number of packets to be sent to the sink node. Relative distance-based protocol (RDBF) [17] uses a fitness function to decide which node will forward the packet towards the sink. Energy efficient routing protocol (EUROP) [18] introduces a layering concept, which divides the sensor nodes into different layers based on depth information. Delay sensitive depth based routing (DSDBR) [19] is another depth based protocol that uses holding time as a measure to reduce transmission delay.

Clustering is a well-known technique used for conservation of energy in sensor networks. Such a scheme is proposed in [20]. In this scheme two- and three-dimension architectures are proposed, which divide the network into two or three clusters, where the cluster head is responsible for communication with the sink node. Dynamic User Coordinate System (DUCS) [21] is a distributed clustering scheme that divides nodes into different clusters; most nodes are limited to communication within the cluster, whereas the cluster head deals with inter-cluster communication. MUD Client Compression Protocol (MCCP) [22] is yet another protocol that selects cluster heads based on the available residual energy, relative position of the node with the sink, and the energy required by the nodes to the candidate node. LCAD (location based clustering algorithm for data gathering) [23] is another such technique developed for three dimensional UWSN. Nodes are deployed in multi-tier fashion and multiple cluster heads are chosen for communication. A review of these techniques is presented in [21,22,23].

AI based routing algorithms have gained popularity in many fields of networking. Correspondingly many Ai based routing algorithms have been proposed for UWSN. One such algorithm is Low-Energy Adaptive Clustering Hierarchy (LEACH), LEACH-C [24], which is an enhancement of the LEACH algorithm. The focus of LEACH-C is selection of CH. This process is based on simulated annealing mechanisms that provide an optimal solution for selection of CH with maximum energy levels. Similarly, in [25] the same mechanism, in two rounds, is used for selection of a secondary cluster head, which becomes the cluster head when the energy level of the cluster head falls below a certain threshold. LEACH-SAGA [26] is another enhancement of the LEACH algorithm, which is based on a simulated annealing and genetic algorithm. In LEACH-SAGA the clusters are formed by genetic algorithms and simulated annealing. Then, based on distance from cluster center and residual energy, the cluster head is chosen. Application-Specific Low Power Routing (ASLPR) [27] is yet another routing algorithm that employs simulated annealing techniques for enhancing the lifetime of a cluster head. ASLPR simply selects a cluster head with a high energy value for a specific time period, and after time expires, a new cluster head is selected, again based on the available energy value, to prolong the life of the nodes.

Particle swarm optimization (PSO) based techniques are popular for optimization in a variety of computing problems. Similarly, PSO based techniques are available for routing protocols of WSN. One such technique is discussed in [28], which is a two stage approach where in the first stage the cluster head is selected on the basis of available energy value as well as network coverage of the node, whereas in the second stage a routing tree is created that connects the cluster heads with the sink node. Another PSO based protocol is presented in [29]. In this approach, an energy conservation-based routing protocol is proposed, which selects the gateway and relay nodes that have the minimum distance from the sink nodes. Moreover, a fitness function is also proposed for load balancing in the network. Particle Swarm Optimization Reactive (PSOR) [30] is yet another PSO based protocol where optimum path is computed on the basis of energy required to reach the sink node. Particle Swarm Optimization-Energy-Efficient Cluster Head Optimization (PSO-ECHS) [31] is a scheme focusing on the efficient selection of a cluster head. For this selection, PSO-ECHS considers residual energy, distance among the nodes in a cluster, and the distance from a sink node. Similarly, clusters are formed using a weigh function with the same parameters.

Ant colony optimization (ACO) is closely related to PSO and is yet another method for optimization. ACO based routing algorithms are popular in various networks, including WSN. ACOPSO [32] is a hybrid routing protocol that employs both ACO and PSO. In this scheme ACO is used for path selection by constructing a shortest path spanning tree. This is followed by PSO based path selection, where inputs are the outputs produced by the ACO. LATWSN [33] is a routing protocol developed by the ACO technique. LATWSN tries to minimize the energy consumption by selecting the node in the neighbor list that is nearest to the destination. LEACH-P [34] is an ACO based extension of LEACH protocol where the probability of energy utilization is calculated while selecting the next hop neighbor towards the sink node. IACAEO [35] is another ACO based protocol, which works by decreasing the pheromone value of a node if the energy level falls beyond a certain threshold. The same information is communicated to its neighbor nodes for non-selection of the node in future routing decisions.

**Figure 1:**
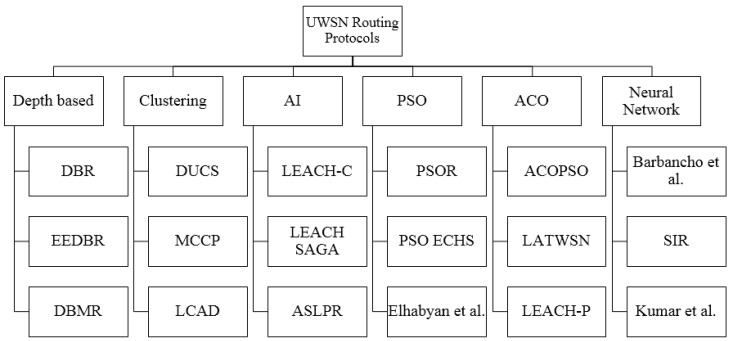
Routing protocols for underwater sensor networks.

Just like ACO and PSO, another popular machine learning technique, neural networks (NN), is also used in WSN for development of an efficient routing algorithm. One such technique is discussed in [36] where a Hopfield NN is used to enhance the working of link state routing protocol. The weighted matrix for initialization of Nneurons and selection of the next node for routing, in this scheme, uses number of hops, load, delay, and bandwidth. SIR [36] is another NN based routing protocol, which is a modification of the classic Dijkstra algorithm for finding optimal routes from a sink to every node. The same algorithm also provides QoS and is used for recording the reading of utility meters. Reference [37] describes another technique where a three stage NN is used to select the cluster head. This is followed by a traditional routing mechanism where the distance between the sink node and NN based cluster head is calculated for selection of an optimal route. These routing protocols categorized in Figure 1.

## 3. Moth Flame Optimizer

Moths are insects that resemble butterflies. To date, about 160,000 species of moths have been identified. Moths are born as larvae, which convert to adult moths in cocoons. The fascinating aspect of moths is the way they navigate at night time. They always travel in the night using moon light. Their method of travelling is known as traverse orientation. In this method, moths always travel towards moon light having a fixed angle towards the moon. This is an effective method for traveling in a straight line. Similar methods can also be used by humans [38,39,40]. Suppose if a human wants to go towards the east and the moon is in the southern side of sky, the person can travel in a straight line if the person keeps the moon to his/her left side while moving. Despite the efficiency of transverse orientation, moths are deceived by artificial light and tend to fly spirally towards artificial light. The reason for this behavior is that transverse orientation works well only if the light source is far away. However, when an artificial light is encountered, moths try to maintain a straight angle towards it. However, since the artificial light is too close, it results in a deadly path for moths. At the same time, it can be said that moths always converge towards light. This is a very useful property, which is exploited mathematically, resulting in the moth flame optimizer (MFO) algorithm introduced in [41]. The future research directions have been discussed in [42,43]. Similarly, future research avenues regarding mobile anchor node assisted localization for WSN are presented in [44]. Localization algorithms of UWSNs are summarized in [45,46].

MFO algorithm moths are treated as candidate solutions where the location of moths in space is considered as a variable for the problem. The moths can fly in either one or two dimensions or in any multi-dimensional space while changing their position. MFO is a population-based algorithm and an m × n moth matrix can be generated from their set of positions, where m is the number of moths and n is the number of variables. A moth array can be created that will store the consequent fitness value of all the moths. This fitness value is the returning value of the fitness function of each moth. Similarly, two matrices are created for flames: the first matrix is like a moth matrix, whereas the other one is like a moth array. Both the matrices have the same degree as the matrices developed for moths. It is important to note that both moth and flame matrices are solutions with the difference being in the way that both are updated in each iteration. Moths are agents moving in the search space, whereas the flame provides the best position that moths have attained yet. We can deduce that the flames are the pins that are dropped by the moths while moving in the search space. Therefore, with each search operation the moth flies around a flame and will update it if it finds a better position. The moth will always find the best solution by adopting this mechanism [39,40,41].

**Figure 2:**
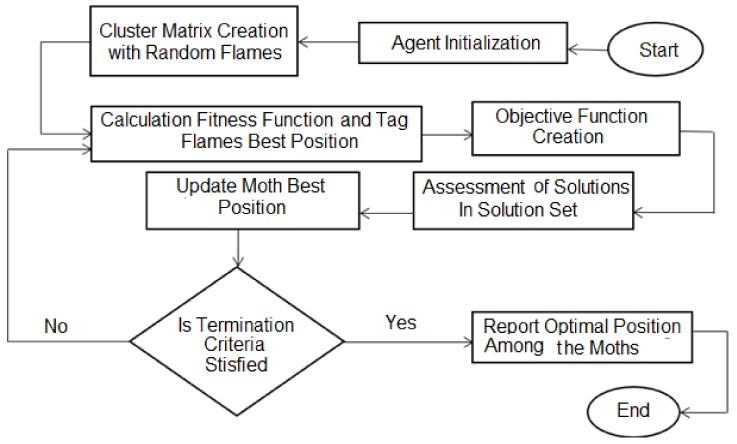
Proposed SOSNET flowchart.

Figure 2 explains the SOSNET algorithm. During the initialization phase, each moth has a random position in an m × n dimensional solution space, whereas fitness values are stored in a moth array. Similarly, a flame matrix and its corresponding array is generated. The flame matrix is used to store the best value of the moth found so far. The moths are moved in the solution space until an optimal solution is found, afterwards the search operation is terminated. This is followed by updating the moth position. The same operation is repeated until the optimal number of flames and correspondingly optimal position of each moth against its flame is obtained.

## 4. SOSNET-Proposed Methodology

CAMONET [38] is an MFO based algorithm for clustering applied in vehicular ad hoc networks (VANET). We believe that the same solution can also be extended to UWSN. CAMONET basically finds the optimal number of clusters required in a given network. Due to its evolutionary capabilities, it finds the best solution for both continuous as well as discrete variable problems. Another promising aspect of this solution is that it is a computationally inexpensive operation that makes it a suitable candidate for UWSN. In [38] we have implemented CAMONET in a VANET environment.

In the current scenario, SOSNET, we create a grid of n × n number of sensors in a geographical area. All the sensors are given a suitable ID in a mesh type network. These nodes are considered as moths. A matrix, based on Euclidean distance, is generated that provides distances of all the nodes. These moths are the basis on which we create our search space. Certain parameters are used for forming this search space: these include dimensions, and lower and upper bounds. This is followed by checking the fitness of moths by using their locations in the search space. This is an iterative process that results in the creation of a fitness matrix; after each iteration, the generated values are stored in this matrix, which are arranged in ascending order. Thus, this matrix provides the lower fitness value of moths. By combining moth position and fitness value we obtain the best score of the flame. This is used for updating the moth position. A linear decreasing factor ‘x’ is used to converge this to an optimal solution. Through this same convergence we can get the optimal number of clusters required for effective communication based on given parameters. The pseudo code of SOSNET in presented in Algorithm 1. 

**Algorithm 1**: Smart Ocean Under Water Sensor Network (SOSNET).1. START 2. Deploy nodes randomly in 2D grid 3. Each node broadcasts its position 4. Generate mesh topology among all nodes while keeping node IDs as vertex. 5. Calculate distance of each node from other nodes and associate these values to the edges in mesh topology. 6. Initialize Moth Position MP and create search space. 7. FOR *i* where *i* starts from 1 to total number of search agents a.FOR *j* where *j* starts from 1 to total number of dimensionsb.Update moth position MP(i,j) = (Upper Bound(i) − Lower Bound(i))/rand( ) + Lower Boundc.END FOR 8. END FOR 9. WHILE Simulation is not end or stall iteration, i.e., 20 iterations a.FOR Moth *i* where *i* starts from 1 to Swarm Sizei.Calculate fitness of each moth position MP (Fitness_of_Moth = FitnessFunction (MP));ii.WHILE list nodes available for clustering is not emptyiii.IF for each Moth cluster fitness is less than Best Solution cluster fitness then allocate that Moth to Best Solution END WHILE
b.END FORc.Sort the fitness values of moths; Sorted_Fitness();d.Sort the population; Population_Sorted(); w.r.t Sorted_Fitness();e.Update the position of best flame obtained so fari.Score of best flame is equal to Sorted_Fitness(1);ii.Position of best flame is equals to Population_Sorted(1,:)
f.Update the position of the moth w.r.t its corresponding flameg.FOR *i* where *i* starts from 1 to total number of search agentsi.FOR *j* where *j* starts from 1 to total number of dimensionsCalculate Distance of the *i*th Moth for the jth flame using distance to flame, which is equal to absolute (Population_Sorted (*i,j*) – MP ( *i,j*))Update the position of moth
ii.END FOR
h.END FORi.IF convergence curve Iteration is equal to convergence curve Iteration-1i.stall iteration++;
j.ELSE stall iteration = 0;k.END IFl.Iteration++; 10. END WHILE 11. Total number of clusters is equal to best solution (from search space) 12. END

Once the clusters are formed, the next phase is to select the cluster head (CH). Selection of the CH depends on many parameters, such as transmission range, residual energy of node, node density, grid size, and load balance factor. Weights have been assigned to all these parameters in the fitness function. This selection is carried out by using a fitness function, which is an important part of our algorithm. Selection of the best CH will increase cluster life time and will ultimately result in saving network energy. SOSNET calculates the fitness value by using the following Equation (1):

(1) $$Fitness=\frac{W1\;\times \;Energy_{Resi}}{\left(W2\;\times \;avg_{dis}\right)\left(W3\;\times \;delta_{diff}\right)}.$$

In the above equation, Energy_Resi_ is the residual energy of the node, whereas avg_dis_ is the average distance towards neighbor nodes and delta_diff_ is the difference. This delta_diff_ is used as load balancing factor (LBF). W_1_ is the assigned weight for energy whereas W_2_ is the weight for average distance and W_3_ is the weight assigned to delta difference. In an ideal scenario, all the clusters should have an equal number of members. However, this is difficult to achieve in a real-world scenario due to changes in sensor positions as a result of ocean currents and other factors. Delta difference is used to calculate the deviation from an ideal degree to a node’s movement from its neighbors and is calculated by following Equation (2):

Delta_Diff_ = ABS (Ideal_Degree_ − Node_Degree_). (2)

Recent research has shown that if the criteria of selection of CHs is static, then there are high chances that a single parameter may bias the fitness function and thus result in selection of an inappropriate CH [21,22,23,38]. To counter this problem, SOSNET uses dynamic assignment of weights to its parameters, based on negative impact on the fitness function, depending on the scenario. It first normalizes the value of each parameter in the range from 0 to 10. Then the deviation, which shows the negative impact of the parameter, is calculated for each parameter from its mean by using the following Equation (3):

Dev (p) = ABS (mean − parameter (p)).(3)

In addition to the above-mentioned equation, another equation is used to penalize outlier parameters, and some penalty is added depending upon the parameter deviation from its mean value. The following Equation (4) is used for this purpose and it computes value for each parameter:

(4) $$w\;\left(p\right)=\;\frac{1}{dev\;\left(p\right)}.$$

The sum of all the weights must be equal to “1”. The fitness value for each node is calculated by using the values and their parameters through Equation (1).

## 5. Experimental Setup

The experimentation was carried out using MATLAB version 2018a (The MathWorks, Inc. 3 Apple Hill Drive Natick, MA 01760, USA), and tested on a corei5, 7th generation system with 8 GB of RAM. Various experiments were carried out using different values of grid size ranging from 500 to 2000 m. Similarly, the number of nodes used in experiments ranged from 20 to 200. The transmission range for each node was also varied from 25 to 200 m. It was assumed that nodes remained in fixed positions or moved very slowly in the presence of water currents.

SOSNET was compared with other state of the art evolutionary clustering protocols: ACO, gray wolf optimization (GWO), and comprehensive learning particle swarm optimization (CLPSO). Table 1 provides the parameters used for simulation.

## 6. Results and Discussion

**Figure 3:**
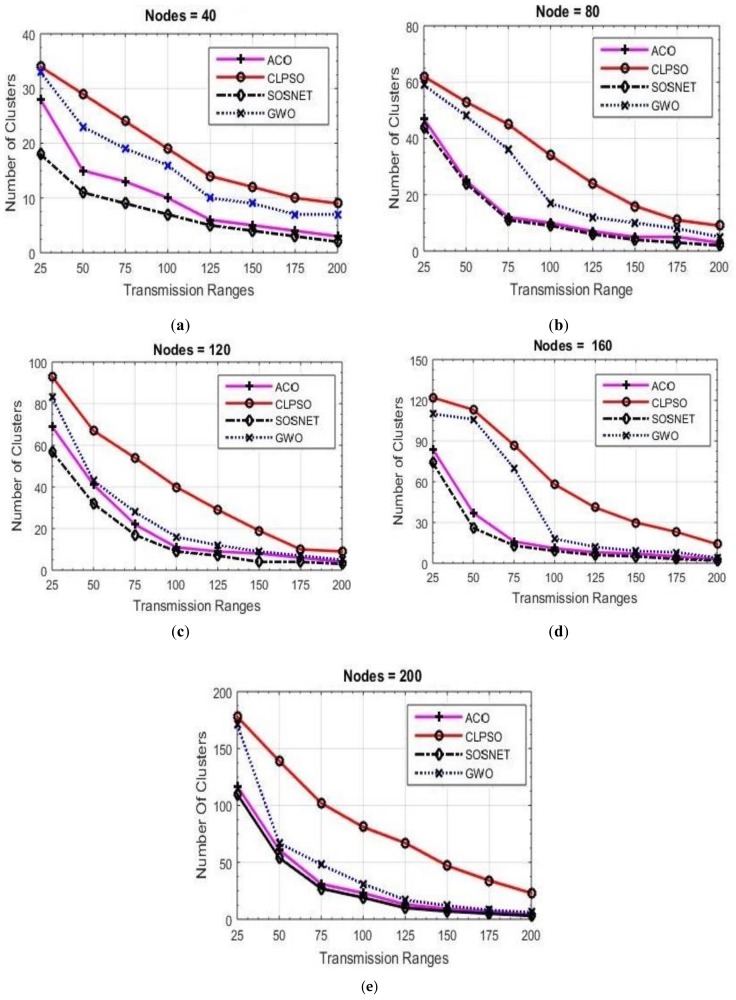
Grid size 500 m × 500 m, nodes 40 to 200.

**Figure 4:**
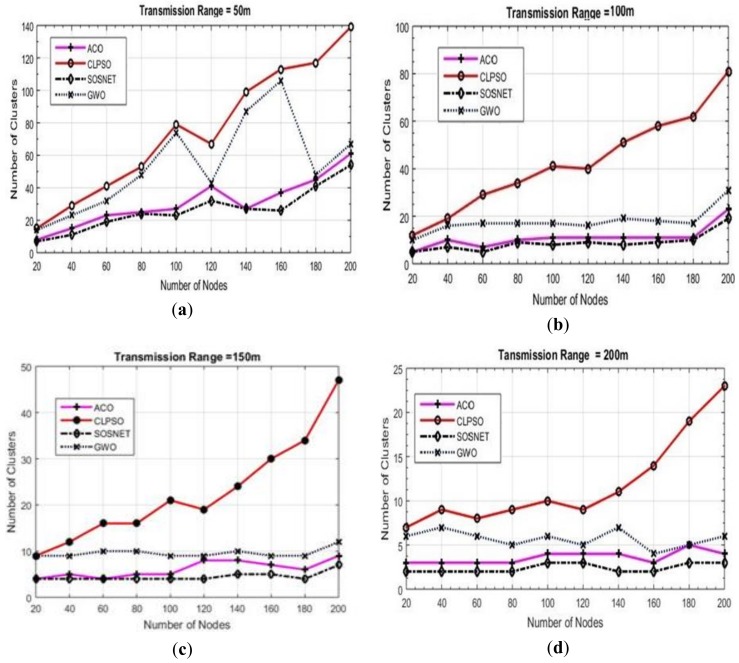
Grid size 500 m × 500 m, transmission ranges 50 to 200.

**Figure 5:**
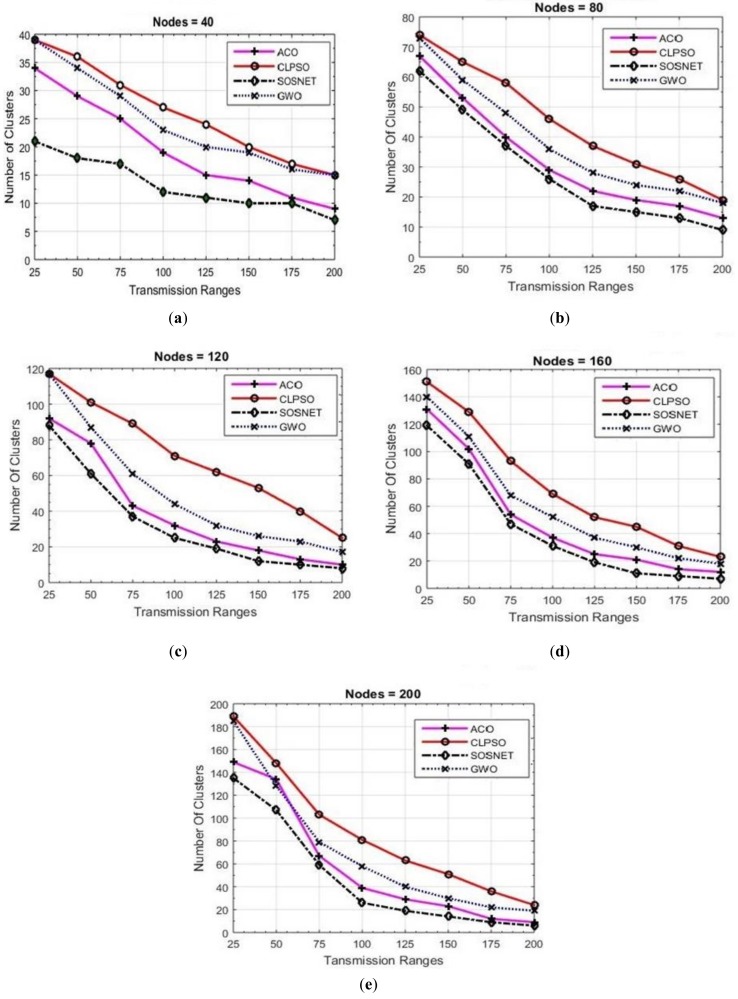
Grid size 1000 m × 1000 m, nodes 40 to 200.

**Figure 6:**
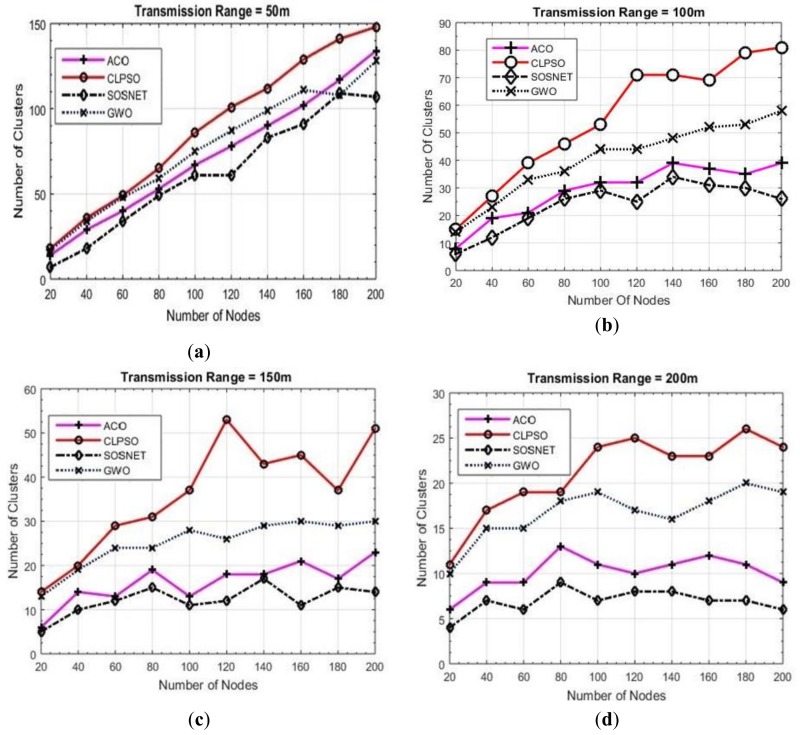
Grid size 1000 m × 1000 m, transmission ranges 50 to 200.

**Figure 7:**
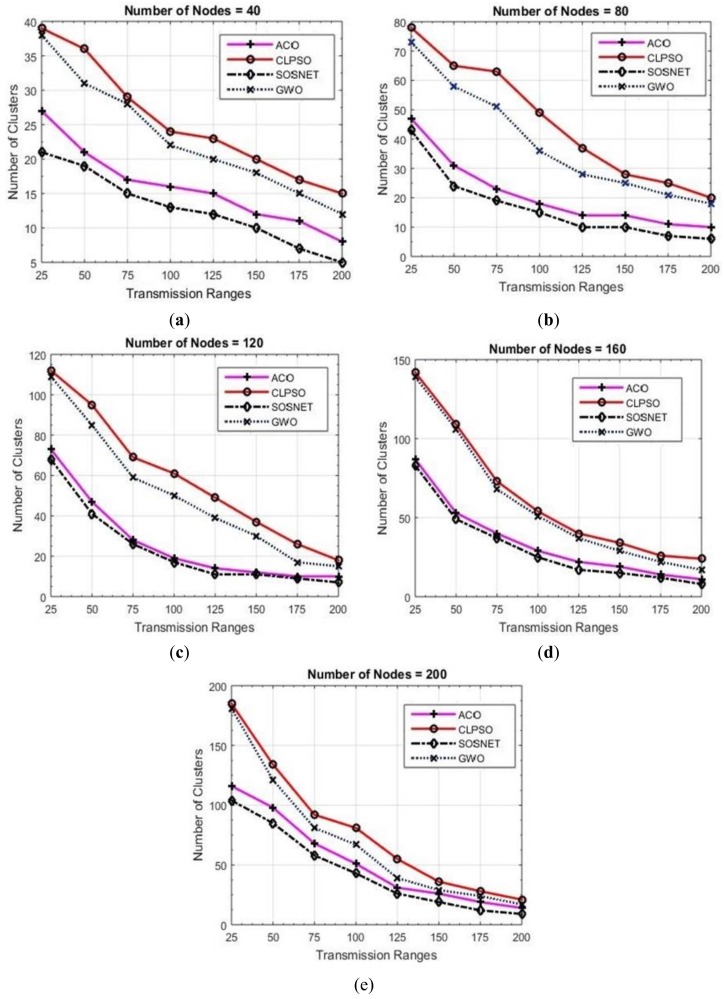
Grid size 1500 m × 1500 m, nodes 40 to 200.

For measuring the performance of each algorithm, fifty-six simulations were performed, and their results are depicted in Figure 3, Figure 4, Figure 5, Figure 6 and Figure 7. Two basic parameters, transmission range of nodes and node density, were evaluated with different values for checking the effectiveness of SOSNET. The results show the flexibility and superiority of SOSNET.

In Figure 3a–e nodes’ transmission range was set in the range of 25 m to 200 m. The grid size was set at 500 m × 500 m and number of nodes in the area was set in the range of 20 to 200 nodes. Figure 3a shows node density of forty nodes with the transmission range set to 25 meters. SOSNET generated only 18 clusters, whereas ACO created 28 clusters, CLPSO 34, and GWO created thirty-three clusters. When the transmission range was increased to 200 meters, SOSNET generated only two clusters in comparison to 3, 9, and 7 for ACO, CLPSO, and GWO, respectively. This is consistent with well-established studies showing that few numbers of clusters will be formed if the transmission range of the nodes are increased. Even when the transmission range was between 25 m and 200 m, SOSNET outperformed all the other algorithms and the results are evident from the figure. Similar kinds of results can be seen when the number of nodes was increased to 40, 80, 120, 160, and 200. This is shown in Figure 3b–e. By viewing the results, it is obvious that SOSNET outperformed all the other algorithms. It created the minimum number of clusters required to work in the given grid size.

### 6.1. Grid Size vs. Transmission Range vs. Number of Clusters

To make matters more concrete another set of experiments was carried out to check the performance of the proposed algorithm. In these experiments we varied the transmission range of the nodes from 50 m to 200 m while keeping the grid size static at 500 m × 500 m and node density was taken as 200 nodes. The results of these experiments are shown in Figure 4. It is evident that even with the varied transmission range SOSNET performed equally well and in all instances gave the best results, whereas ACO was the closest one in these settings.

Even with the grid size increased to 1 km × 1 km SOSNET outperformed all the other algorithms. The results of this scenario are depicted in Figure 5. In this setting, when the node density was set at eighty nodes, SOSNET created 9 clusters. On the other hand, ACO created 13, CLPSO 19, and GWO 18 number of clusters. Clearly, SOSNET was providing a smaller number of clusters. When the node density was increased to 200, ACO created 9 clusters, GWO 19, and CLPSO created 24 clusters. On the contrary SOSNET created only six clusters, which showed that with this grid size, at any number of node density, SOSNET created a smaller number of clusters, thus optimizing the routing problem. It can also be seen that the transmission range had a direct impact on the number of clusters. This is consistent with other studies that show that more clusters are formed if the transmission range is low, since there tend to be fewer nodes in a cluster. If the range is increased, the nodes will find farther nodes as well, resulting in a fewer number of clusters.

To further analyze the performance of SOSNET, the algorithm was experimented with different transmission ranges of the nodes while keeping the grid size and node density at a fixed value, i.e., 1 km × 1 km and 200 nodes, respectively. The results, as shown in Figure 6, indicate that SOSNET was a perfect solution in the given situation and provided optimal results.

Simulations were also carried out while increasing the grid size to 1500 m × 1500 m. The results of this setting are shown in Figure 7. It can be observed from Figure 6a that with lower transmission range all the algorithms except SOSNET provided similar results, whereas SOSNET provided optimal results, which shows the efficiency of the algorithm. It can also be observed that with the increasing number of nodes the performance of SOSNET also improved. For example, with the node density set to 200, SOSNET generated 9 clusters, whereas ACO was the second best with 14 clusters, GWO created 17 clusters, and CLPSO generated 21 clusters.

**Figure 8:**
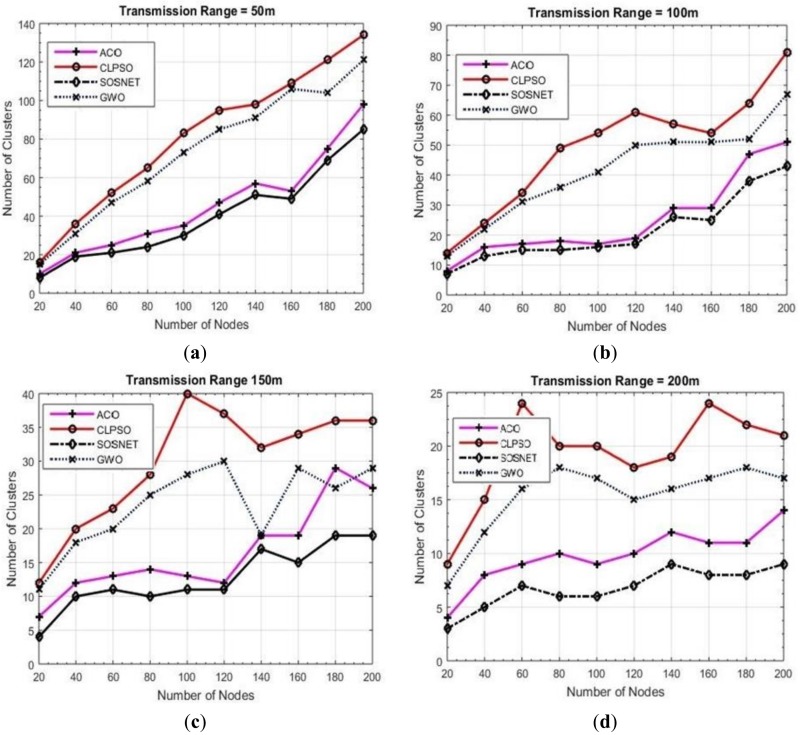
Grid size 1500 m ×1500 m, transmission ranges 50 to 200.

As with the previous two scenarios, SOSNET was again evaluated with the varied transmission range from 50 m to 200 m and keeping the grid size at 1500 m × 1500 m. The results are depicted in Figure 8. It is interesting to note that there was a huge performance difference between SOSNET and the other algorithms when the transmission range of the nodes was set to 200 m in the given setting. This shows that with the increasing transmission range the performance of SOSNET improved further and it created very few numbers of clusters.

**Figure 9:**
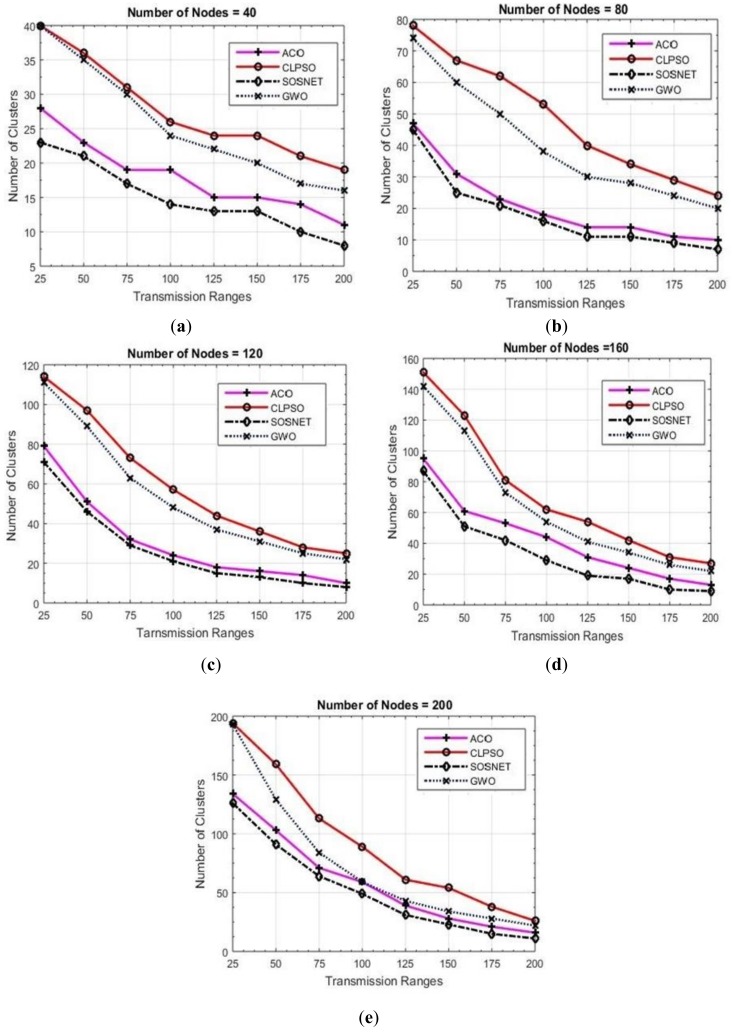
Grid size 2000 m × 2000 m, nodes 40 to 200.

Similar kinds of results were observed when the grid size was enhanced to 2 km × 2 km. The results of this grid size are shown in Figure 9. In this setting, with the transmission range set to 100 m and node density set at 160 nodes, both CLPSO and GWO performed poorly, creating 27 and 22 clusters, respectively, whereas ACO created 13 clusters. However, SOSNET performed excellently by generating only nine clusters. In the extreme situation where transmission range was considered as 200 m and node density was set to 200, SOSNET generated 11 clusters, whereas the closest one, i.e., ACO, generated 16 clusters. The same results were observed when various parameters were changed, such as grid size, transmission range, and number of nodes. This proves that SOSNET was the best solution and provided the optimal number of clusters in any given setting.

**Figure 10:**
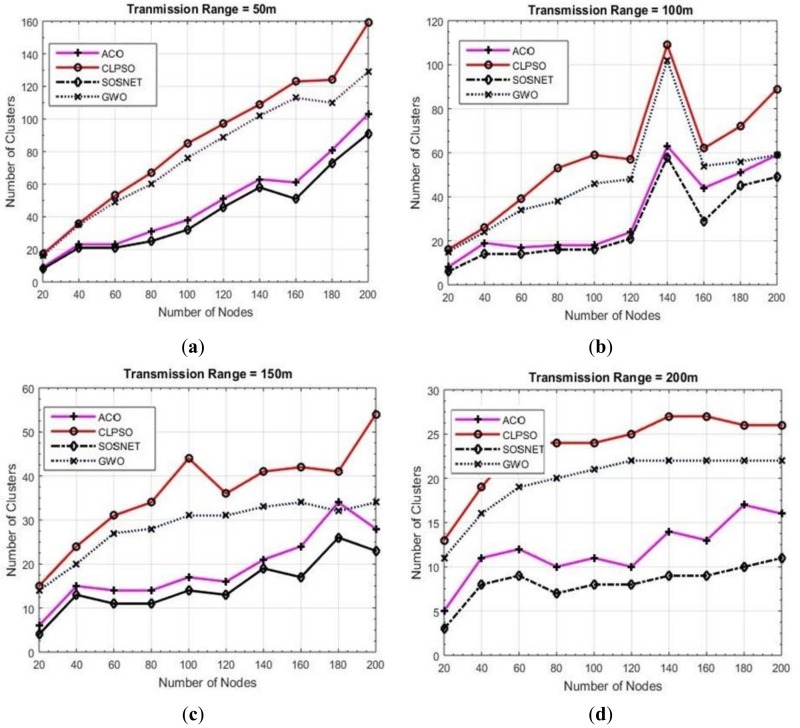
Grid size 2000 m × 2000 m, transmission ranges 50 to 200.

The same results were also observed with the transmission range set to 50, 100, 150, and 200 m while keeping other settings constant at 200 nodes and 2 km × 2km. It can be clearly observed from Figure 10 that SOSNET’s performance was best among these protocols. This shows that SOSNET gave the best results in any given scenario.

**Figure 11:**
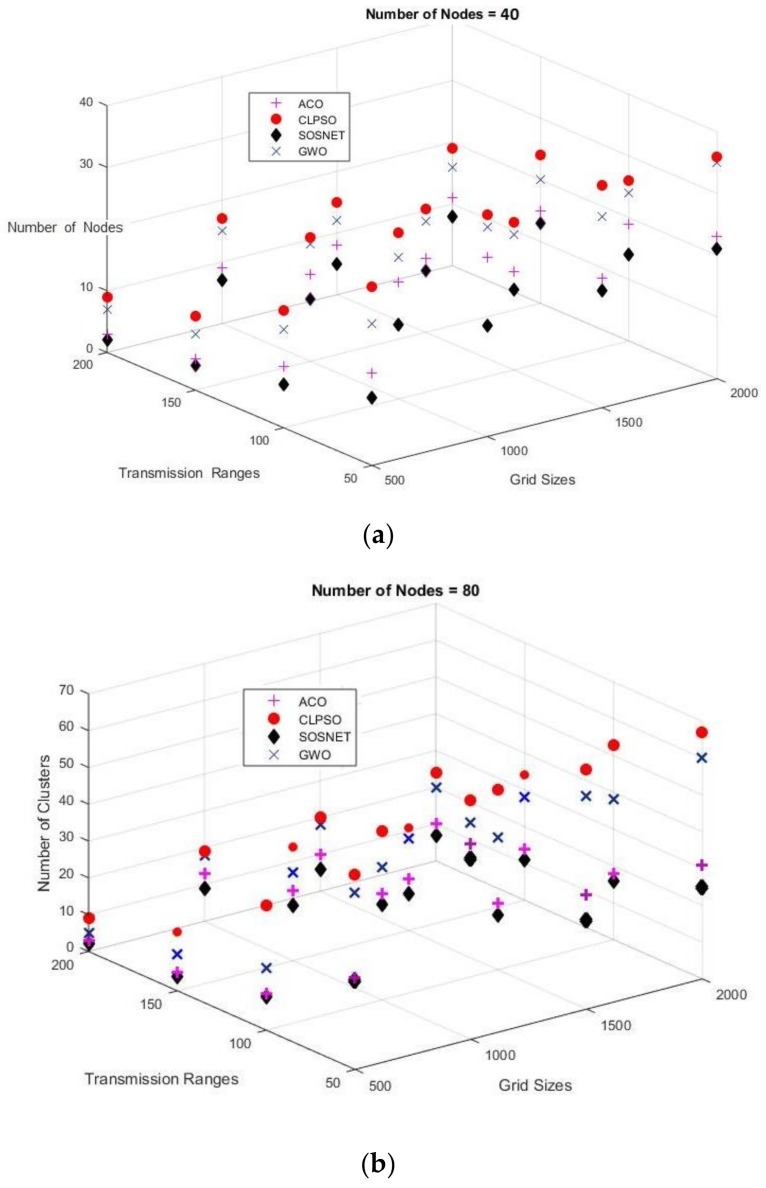
3D grid, transmission range 50 m to 200 m, grid sizes from 500 m to 2000 m for 40 and 80 nodes.

In order to evaluate SOSNET in a more realistic real-world scenario, a final set of simulations was also run on a 3D grid with a transmission range of 50 m to 200 m and a grid size of 500 m × 2000 m with the node density set to 40 and 80 nodes. The results are depicted in Figure 11. It shows that even in this scenario, SOSNET performed better than the other algorithms.

### 6.2. Load Balance Factor

**Figure 12:**
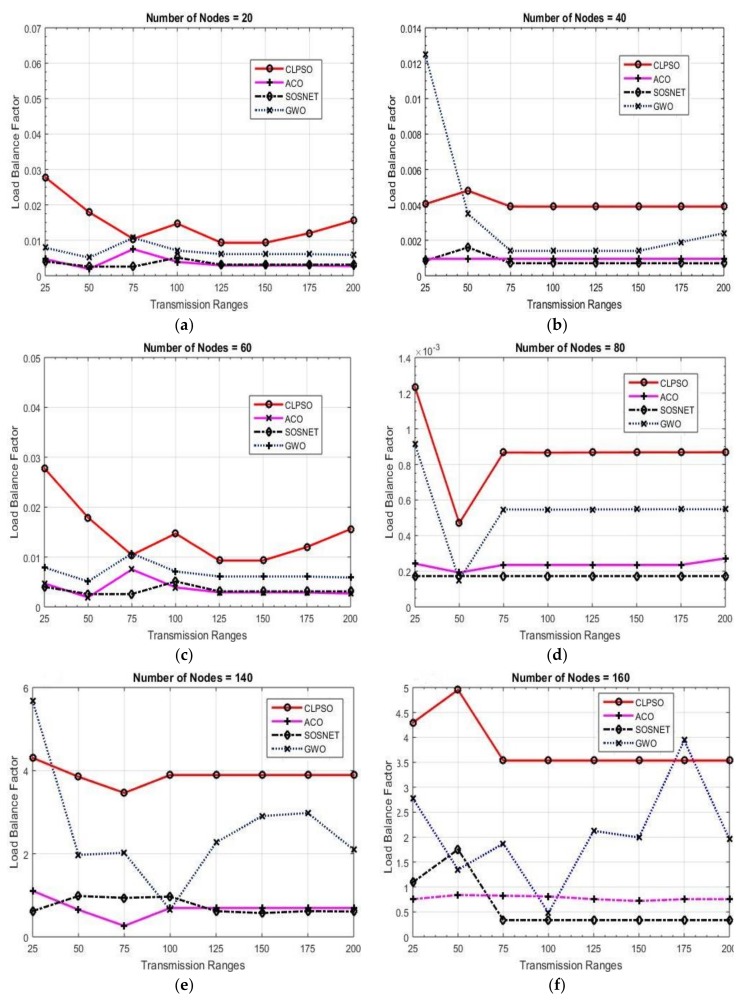
Load balancing factor for 1000 m × 1000 m grid and transmission ranges 25 m to 200 m.

As mentioned above, it is not possible to have an equal number of nodes in each cluster. It is important to calculate the load on each CH. LBF is also calculated for each CH by following Equation (5):

(5) $$LBF=\;\frac{1}{n_{c\;}x\;\sum_{i}{\left(x_{i\;}-u\right)}^{2}}$$

where *n_c_* is the number of clusters, *x_i_* is the cluster’s cardinality, and –*u* is the average number of neighbors of a CH. Figure 12 shows the LBF calculated for a grid size of 1000 m × 1000 m; the transmission range is taken from 25 m and the node density ranges from 20 to 200 nodes. It can be observed that SOSNET performed well when the neighbors’ numbers reached the threshold value in terms of load balancing in the network.

By looking at Figure 12, some general observations can be made. When the transmission range was increased, a fewer number of clusters were formed. This implies that the number of clusters is inversely proportional to the transmission range. On the other hand, an increase in grid size resulted in a higher number of clusters, effectively showing that both these were proportional to each other. Another observation can be made while observing the distance between nodes and grid size. The distance between the nodes and grid size were directly proportional to each other.

The above discussion and results prove that SOSNET provides optimal result in UWSN and make it a promising candidate of routing algorithms in this scenario. SOSNET works efficiently, not only in the presence of dense traffic, but it also performs equally well in large network sizes and in any transmission range, which makes it an ideal choice for a routing algorithm that can be used in UWSNs.

## 7. Conclusions

In the current research work, we have proposed an efficient algorithm SOSNET for UWSN. SOSNET is a scalable and efficient protocol that utilizes the MFO technique to work in a search space and selects the optimal number of clusters for routing. SOSNET is an evolutionary algorithm that works iteratively in a given search space. As the total number of clusters required are decreased, the routing cost of the packets is also reduced. It provides a near optimal number of clusters required for routing, and this ultimately results in decreasing the routing cost as well as the conservation of energy in the nodes.

To check the effectiveness of the proposed algorithm, different simulations were executed. The algorithm was tested with various levels of node density; similarly, the algorithm was tested by varying the transmission range of sensor nodes. The results prove that SOSNET is an ideal solution that can be adopted for routing in the said networks. SOSNET is compared with other well-known evolutionary algorithms, i.e., GWO, CLPSO, and ACO, and the results show the superiority of the SOSNET.

## Figures and Tables

**Table 1:** Simulation parameters.

Parameters	SOSNET	ACO	GWO	CLPSO
Population Size	100	100	100	100
Maximum Iterations	150	150	150	150
Simulation Runs	10	10	10	10
Inertia Weight	0.90	-	0.694	0.694
Evaporation Rate	-	0.5	-	-
C1­_1_	2	2	2	2
C2_2_	2	2	2	2
Simulation Area	500 m^2^, 1000 m^2^, 1500 m^2^, 2000 m^2^	500 m^2^, 1000 m^2^, 1500 m^2^, 2000 m^2^	500 m^2^, 1000 m^2^, 1500 m^2^, 2000 m^2^	500 m^2^, 1000 m^2^, 1500 m^2^, 2000 m^2^
Number of Underwater Sensors Nodes	20 to 200	20 to 200	20 to 200	20 to 200
Interval B/W Nodes	+20	+20	+20	+20
Transmission Ranges	25 m to 200 m	25 m to 200 m	25 m to 200 m	25 m to 200 m
Nodes Positions	Fixed	Fixed	Fixed	Fixed
Minimum Distance B/W Nodes	2 m	2 m	2 m	2 m
Maximum Distance B/W Nodes	5 m	5 m	5 m	5 m
W_1_ (weight for 1st Objective Function)	0.5	0.5	0.5	0.5
W_1_ (weight for 2nd Objective Function)	0.5	0.5	0.5	0.5
